# Immigration, Local Dispersal Limitation, and the Repeatability of Community Composition under Neutral and Niche Dynamics

**DOI:** 10.1371/journal.pone.0046164

**Published:** 2012-09-24

**Authors:** Dexiecuo Ai, Philippe Desjardins-Proulx, Chengjin Chu, Gang Wang

**Affiliations:** 1 School of Life Sciences, Lanzhou University, Lanzhou, China; 2 Theoretical Ecosystem Ecology Laboratory, Université du Québec à Rimouski, Rimouski, Canada; 3 Quebec Center for Biodiversity Science, McGill University, Montréal, Canada; 4 College of Engineering, University of Illinois at Chicago, Chicago, Illinois, United States of America; CNRS, University of Montpellier II, France

## Abstract

Repeatability of community composition has been a critical aspect for community structure, which is closely associated with community stability, predictability, conservation biology and ecological restoration. It has been shown that both immigration and local dispersal limitation can affect the community composition in both neutral and niche model. Hence, we use a spatially explicit individual-based model to investigate the potential influence of immigration rate and strength of local dispersal limitation on repeatability in both neutral and niche models. Similarity measures are used to quantify repeatability. We examine the repeatability of community composition among replicate communities (which means the same community repeats many times), and between niche and neutral replicate communities. We find the correlation between repeatability and immigration rate is positive in the neutral model and an inverted unimodal in the niche model. The correlation between repeatability and local dispersal distance is positive in the niche model and negative in the neutral model. High repeatability between niche communities and neutral communities is observed with high immigration rates or when high local dispersal distance appears in the niche model or low local dispersal distance in the neutral model. Our results show that repeatability of community composition is not only dependent on the types of community models (niche vs. neutrality) but also strongly determined by immigration rates and local dispersal limitation.

## Introduction

Repeatability of community composition is a key property of community stability and predictability [1]–[5], which also plays a major role in conservation biology and ecological restoration and can be used as the foundation for regional conservation planning [6]. For example, in many instances it is desired that similar assemblages arise after large disturbances, e.g. in population management and conservation [7]. Here, we define the repeatability as the similarity of community composition among replicate communities which means that the same community repeats many times, assembled under identical conditions [7].

The repeatability of community composition is one of the important aspects of community assembly [7], like species diversity and relative species abundance distribution [8]–[11]. The factors influencing the community composition are different between classic niche and neutral theory. In classic niche theory, community composition is determined by species-specific difference and heterogeneous environment [11]–[14]. However, in neutral theory, it is determined by ecological drift and immigration rate [8], [15], [16]. In addition, more studies have showed that incorporating immigration and local dispersal limitation with niche theory can preferably explain community composition; meanwhile these two factors also play important roles in neutral theory [17]–[19].

It has well demonstrated that immigration is a potential factor affecting community similarity [20], [21]. Without immigration, the niche theory is insufficient to explain the species richness observed in nature, because the number of coexisting species in classic niche communities will be no greater than the number of limiting resources [11], [22]–[24]. However, high immigration rates will compensate for a low species persistence through mass- or rescue-effects [25]–[27]. Loreau and Mouquet [18] investigated the influence of immigration from a regional pool on a plant community governed by competition for space and found that immigration could have a huge effect on local species diversity in competitive communities. In neutral theory, species diversity is maintained by a stochastic balance between extinction and immigration [8], [9], [17]. In the absence of immigration and speciation, local neutral communities will collapse to a single species [28]. Thus, immigration is a critical component for both the niche and neutral models.

Closely associated with immigration, dispersal limitation is another important factor influencing the community composition [19], [29]. In Hubbell's neutral model [8], the dispersal limitation is implemented by limiting the immigration rate from the outside and it occurs between local communities or between local community and metacommunity. Here, we define local dispersal limitation (hereafter simply referred to as dispersal limitation) as spatially limited dispersal in local communities [19], [29], [30]. Dispersal limitations determine patterns of distribution among species whose ecological abilities are predicted to be largely equivalent [19], [29]. In spatial niche models, in contrast, the dispersal limitation acts in conjunction with species specific environmental conditions to determine the distribution of species [31], [32]. Chave et al. [19] found that dispersal limitation plays an important role in species distribution for both neutral and niche models.

Since repeatability is an important aspect of community assembly and immigration and dispersal limitation play significant roles in community composition, we investigate in detail the effects of immigration rate and strength of dispersal limitation on repeatability of community composition among replicate communities in both neutral and niche theories using an individual-based spatially explicit model. The neutral model is akin to Gravel et al. [33], while the environment is heterogeneity which remains constant in all simulations and each species has an optimal environmental condition in niche model. The immigrants come from a common regional species pool. The regional species pool is important for community restoration [34] and the first step of ecological restoration is the re-establishment of plant communities at degraded sites through immigration from the regional species pool [34]–[36]. We examined three different kinds of dispersal limitation: four nearest-neighbors dispersal, the Gaussian dispersal kernel and global dispersal. In order to investigate the repeatability of community composition at the species level (species present or absence) and at the individual level (the abundance of each species), we use the Jaccard and Bray-Curtis similarity indexes [37]–[39], respectively.

We examine the repeatability among neutral replicate communities and niche replicate communities separately. As higher immigration rates lead to a high probability that species found at the regional scale will also be found in the local community [8], [40], repeatability among neutral replicate communities will increase with immigration. In contrast, niche models often rely on deterministic processes and species adapted to the environmental conditions can exist in closed communities. Our first prediction is that, as immigration rate increases, the repeatability among neutral replicate communities will increase while the repeatability among niche replicate communities will decrease. However, much higher immigration should increase the repeatability in both cases because of the high number of individuals coming from the same pool. In our models immigration and dispersal have very different effects. Immigration brings individuals from the regional pool to the local community, while dispersal is the movement of individuals within a local community. Our second prediction is that repeatability among replicate communities will increase as dispersal limitation decreases in both neutral and niche models. It is simply because long dispersal distances leads to homogeneous species distributions in neutral models and species are more likely to find their favorite habitat in niche models. We also investigate the effect of immigration rates and dispersal limitation on repeatability between neutral and niche replicate communities. This is necessary because we can control the immigration or dispersal distance to recover the community from damage, even if we have no idea about the mechanisms maintaining species diversity. In addition, at small and intermediate spatial scales, it is more challenging to distinguish the effects of niche versus neutral processes on community composition [41], [42].

## Results

### The influence of immigration rate

**Figure 1:**
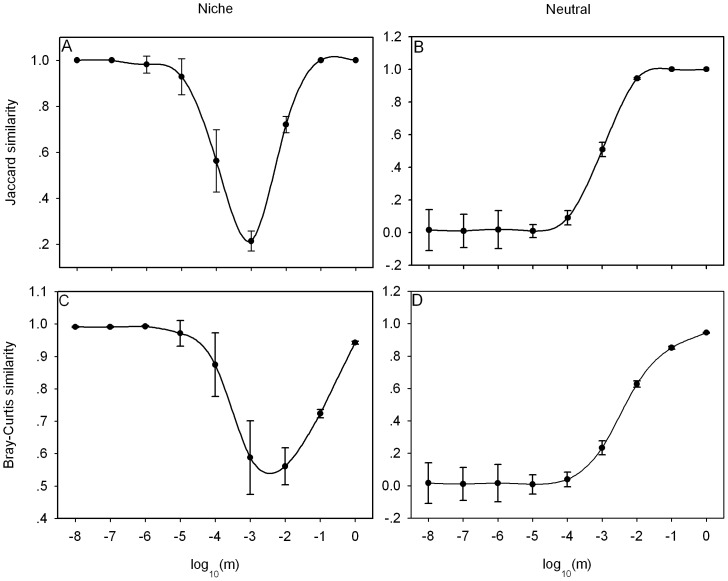
The effect of immigration rates on repeatability of community composition in the different models. A logarithmic scale is used for *m*. Error bars show the standard deviation. Data points are the mean values for 20 replicates.

The models differ substantially in repeatability. Fig. 1 illustrates the effect of different immigration rates on community repeatability under niche and neutral model. We found that repeatability is average among replicate communities by paired comparison. In the niche model, repeatability first decreases to a critical point, after which it increases (Fig. 1A, 1C). However, for Jaccard similarity, the lowest point appears at *m* = 10^−3^, whereas it occurs at *m* = 10^−2.5^ for Bray-Curtis similarity. With very low and very high immigration rates, Jaccard similarity is 1.0 (Fig. 1A), while Bray-Curtis similarity remains nearly constant at low immigration rates (Fig. 1C). With very low and very high immigration rates, the Jaccard similarity of neutral communities is nearly constant (Fig. 1B). The lowest and the highest points happen at *m* = 10^−5^ and *m* = 0.1, respectively. Between these two extremes, Jaccard similarity obviously increases with immigration rate. However, the Bray-Curtis similarity first remains constant and then increases as immigration rate increases (Fig. 1D). The break point appears at *m* = 10^−5^.

**Figure 2:**
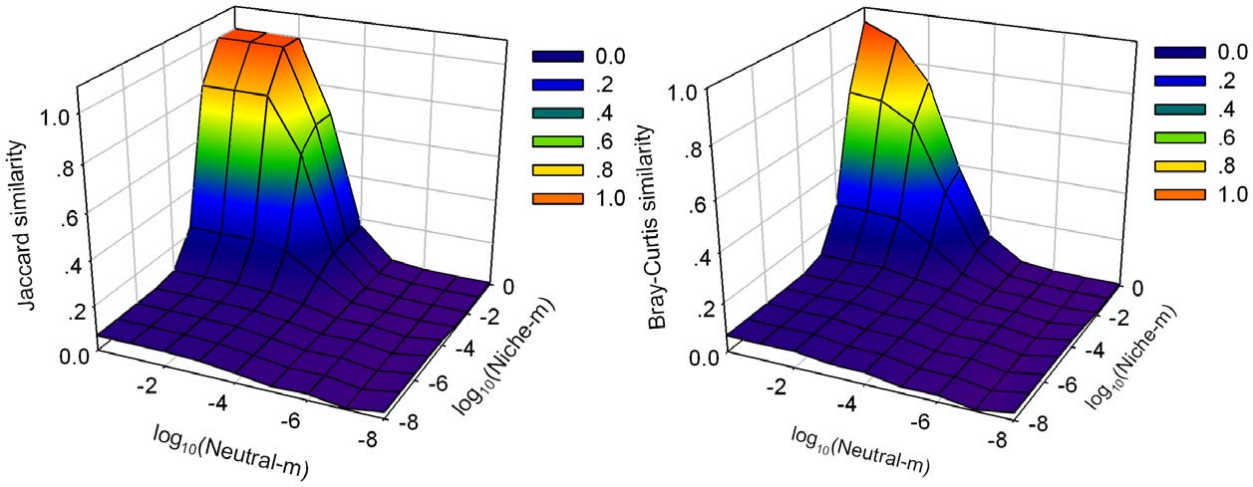
The repeatability between niche and neutral communities under different immigration rates. Neutral-*m* indicates the immigration of neutral communities and niche-*m* represents the immigration of niche communities, both on a logarithmic scale. The data points are means for 400 replications.

The repeatability between neutral and niche communities are affected by immigration rates (Fig. 2). A high immigration rate is needed to generate high repeatability between neutral and niche communities. The highest Bray-Curtis similarity is less than 1.0, while the highest Jaccard similarity is 1.0. For a given immigration rate and community type, increasing the immigration of another community type can increase the repeatability.

**Figure 3:**
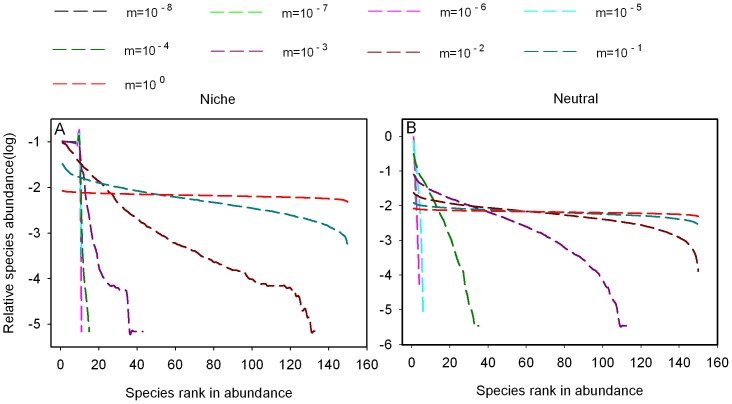
The influence of immigration rates on the equilibrium relative species abundance curves for the different models. Notice that the curves for m = 10^−8^ and 10^−7^ overlap.

The rank-abundance curves become flat as immigration rate increases in both neutral and niche communities (Fig. 3). For a given immigration rate except *m* = 1, the relative species abundance curve of niche communities is steeper than for neutral communities. When *m* = 1, the rank-abundance curves are similar in flat both at niche and neutral model.

### The influence of dispersal limitation

**Figure 4:**
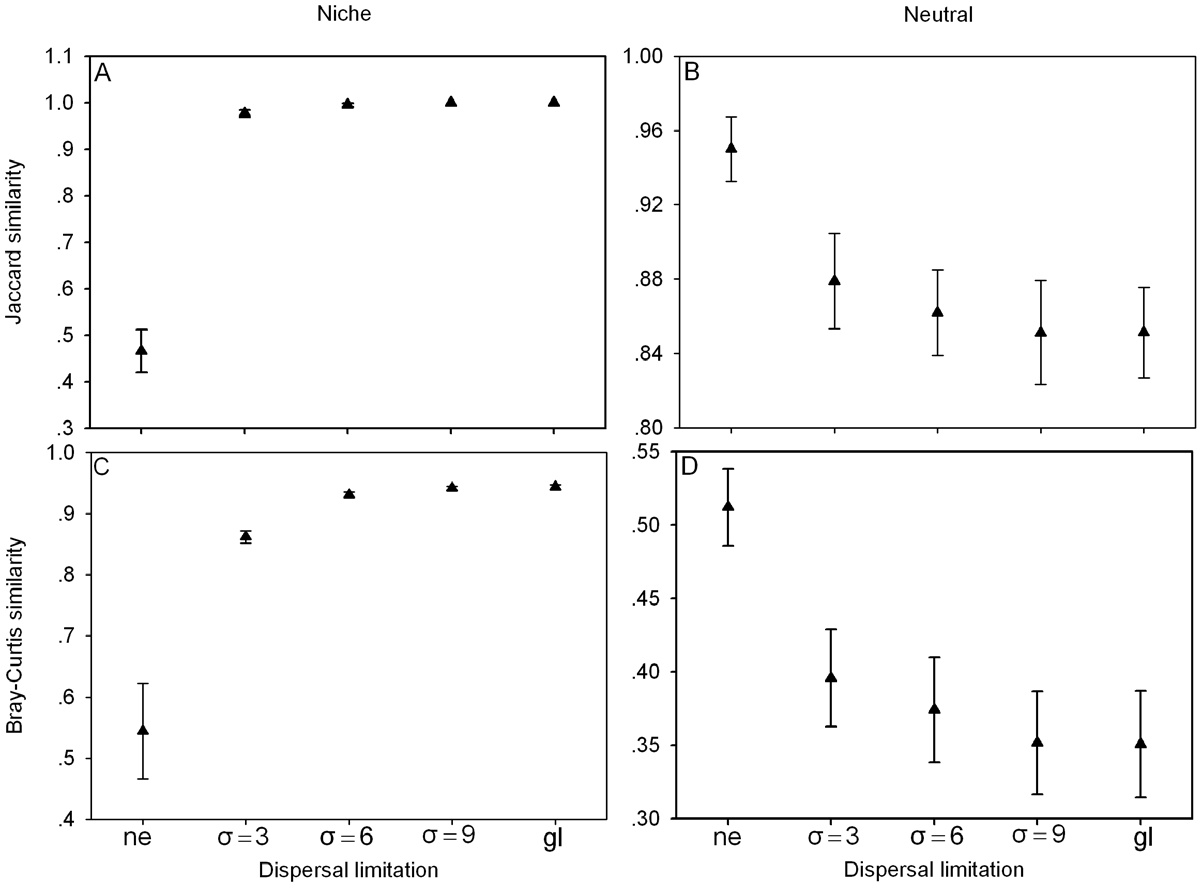
The effect of dispersal limitation on the equilibrium repeatability in the different models. Error bars show standard deviations. *ne* denotes the four nearest neighbor dispersal, *gl* denotes global dispersal, and *σ* is the mean dispersal distance in the Gaussian dispersal kernel. For all runs *m* = 0.005. The data points are the means for 20 replicates.

The effect of dispersal limitation on community repeatability varies between niche and neutral models and the repeatability is averaged among replicate communities by paired comparison in each model (Fig. 4). The repeatability of niche communities increases as dispersal distance increases (Fig. 4A, 4C). Jaccard similarity approaches 1.0 quickly and then remains constant, whilst the Bray-Curtis similarity first increases fast and then increase slow. For neutral communities we find a negative relationship between repeatability and dispersal distance (Fig. 4B, 4D).

**Figure 5:**
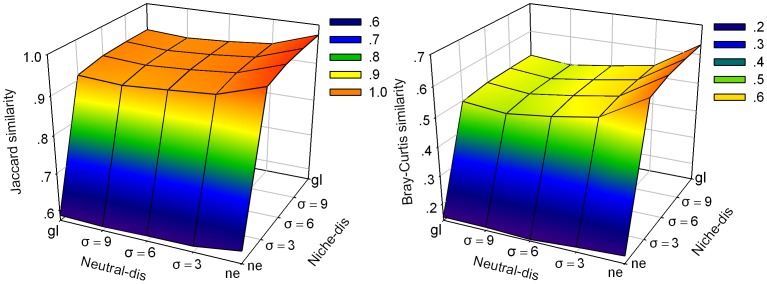
The equilibrium repeatability between niche and neutral communities under different dispersal limitations. Neutral-*dis* indicates the dispersal distance of neutral communities and niche-*dis* represents the dispersal distance of niche communities. The default parameters are the same as in Fig. 4.

The influence of dispersal limitation on repeatability between neutral and niche communities is shown in Fig. 5. When the dispersal distance is large in the niche model and is small in neutral model, the repeatability between them will be large. For a given dispersal distance in the niche model, increasing the dispersal distance in neutral communities decrease repeatability. In contrast, for a given dispersal distance in the neutral model, there is positive correlation between dispersal distance in niche community and repeatability. The highest Jaccard similarity between niche and neutral communities is 1.0, while the highest Bray-Curtis similarity is less than 0.7.

**Figure 6:**
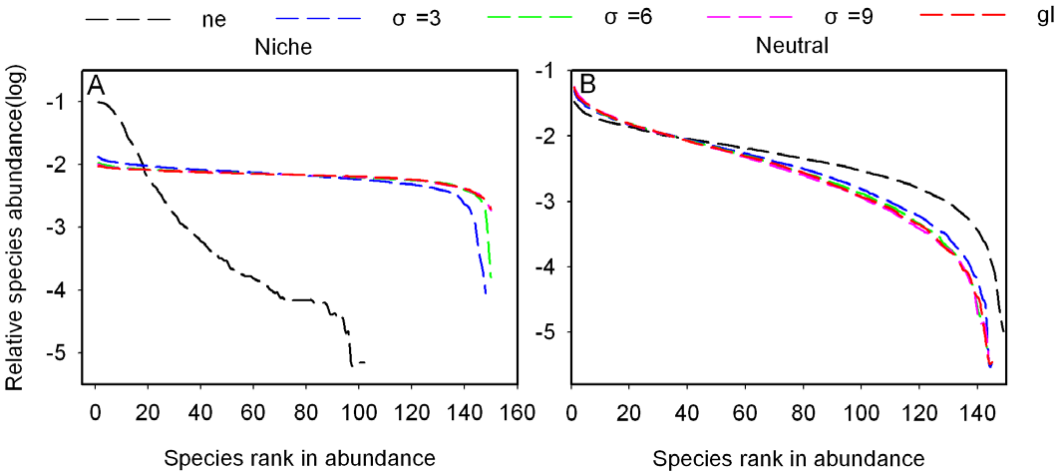
The influence of dispersal limitation on equilibrium relative species abundance curves for the different models. The default parameters are the same as in Fig. 4.

The effect of dispersal limitation on relative abundance distributions differs between niche and neutral model (Fig. 6). In the niche model, rank- abundance curves become more flat as dispersal distance increases (Fig. 6A), while they become steeper as dispersal distance increases in neutral communities (Fig. 6B).

## Discussion

We investigated the influence of immigration rates and dispersal limitation on repeatability in neutral and niche communities separately and between neutral and niche communities together. However, we do find that repeatability differs significantly under niche and neutral models.

### For immigration rate

Our results reveal that the neutral theory can produce high repeatability, provided that immigration rate from regional species pool is high. According to the neutral theory, differences in relative abundances are primarily explained by stochastic processes [8]. In addition, species diversity is maintained by the balance between extinction and immigration at the local community scale. In the case of high immigration rates, the local community contains a large number of immigrants and does not differ much in community composition from the regional pool. In contrast, when immigration is restricted, stochastic processes have a strong influence on community composition and repeatability is small. Increasing immigration rates decreases the rate of divergence in diversity among communities [16], so repeatability increases. However, the Jaccard index focuses on species present or not while the Bray-Curtis focuses on relative abundance level, so the Jaccard similarity reaches 1.0 when species composition in two local communities are the same but the Bray-Curtis similarity can hardly reach such high value (Fig. 1B, D).

In niche models, with low immigration rates, some species which can adapt the environmental condition could be maintained in all replicate communities and, as species diversity is mainly influenced by deterministic factors [43], [44], so repeatability can be quite high (Fig. 1A, 1C). Increasing immigration leads to an increase in the number of individuals from the regional species pool [45]–[49]. However, at interdediate migration rate, the individuals coming from regional species pool are limited and the migrants as inferior competitors can be maintained in the communities. According to Tilman [50], propagules must survive long enough to become reproductively successful adults while using the resources left unconsumed by established species.These inferior competitors have difficulties to establish themselves successfully. Added to stochasticity, this leads to lower repeatability of community composition. At much higher immigration rates, although the competitive exclusion is more intense, the high number of immigrants from the regional pool compensates for competitive exclusion and the abundances of all species are distributed evenly in all replicate communities, so repeatability increases.

High repeatability is possible between neutral and niche communities when high immigration rates are introduced (Fig. 2). The reason is that high immigration rates increase the probability that a species will appear in both types of communities and abundance of species are more evenly (see Fig. 3). Nevertheless, the highest Bray-Curtis similarity is still lower than the highest Jaccard similarity, because Bray-Curtis index is most sensitive to differences in the relative species abundance [38], [39] and some specific species dominate the niche communities even with higher immigration rates. The species adapted to the environmental condition are superior competitiors and can outcompete immigrants, so the relative abundance curves are steeper in niche model than neutral model in which all species have the same competitive ability for a given immigration rates (Fig. 3). Individual death is random in both models, when *m* = 1 all dead individuals are replaced by immigrants from the regional species pool, so rank-abundance distribution are similar in both models.

### For dispersal limitation

In neutral models, patterns of species distributions are strongly determined by dispersal limitation [19]. In our neutral model, repeatability of community composition decreases as dispersal distance increases, contrasted with the prediction. Under local dispersal, propagules land on sites near their parents [51] and local competition enhances species richness [52], while the propagules could land on all sites of the community under global dispersal and stochastic competitive exclusion increases, which results in the decrease of repeatability (Fig. 4B, D). Interspecific competition will be decreased by the life-history niche difference, because, on average, only those propagules which adapt the environmental condition can land on the empty sites successfully. In our niche model, species with high dispersal have high probability to occupy the favorable empty sites, and then they can escape stochastic extinction and the number of species increases (Fig. 6A), leading to an increase in repeatability (Fig. 4A, 4C).

The repeatability between niche and neutral communities is high only if the dispersal distance of the species in the niche model is large and if it is small in the neutral model (Fig. 5). This may be because increasing dispersal distance results in increasing species richness and reducing the difference among species relative abundance in niche model (see Fig. 6A). On the contrary, species richness decreases and difference among species relative abundance increases as dispersal distance increases in neutral model (see Fig. 6B). These results are consistent with Chave et al. [19], who found that species richness was higher under local dispersal than under longer-distance dispersal.

In this study, we show that repeatability of community composition correlates not only with niche and neutral model but also with immigration rates and dispersal limitation. Understanding how immigration and dispersal limitation affect repeatability of community composition could contribute to the restoration ecology.

## Methods

### General framework

To simulate the repeatability of local communities, we implement an individual-based spatially explicit model. The local community is defined as a two-dimensional lattice. It is divided in *L×L* sites and assumed to be a torus. Simulations start with *M* species and the abundance of each species is *(L×L)/M*. All individuals are randomly placed on the landscape and each site can be inhabited by a single individual. Our model follows the zero-sum dynamics as neutral theory [8]. At each time step, each individual died with probability *d*, and the empty sites will be occupy either by the immigrants from regional species pool with probability *m*, or by individuals from the same local community.

### Neutral model

In neutral model, all individuals have the same demographic attributes. The fecundity of all species is infinite which means each individual prepares to occupy the site if it is necessary [19]. A dead individual is replaced by a new individual with probability *R_i_* [33]:
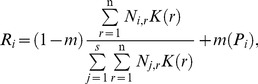
(1)where *r* is the distance between two sites and *σ* the mean dispersal distance. *N_i,r_* is the group of individuals of species *i* within some distance *r* from the recruited site. Parameter *m* is the probability that a recruit is an immigrant coming from the regional species pool. *K(r)* is the dispersal kernel. *P_i_* is the relative abundance of species *i* in the regional species pool. The first term is the contribution of the local population of species *i* to the propagule supply. The second term is the contribution of the regional species pool of species *i* to the propagule supply.

We examined three different kinds of dispersal limitation: global dispersal, four nearest-neighbors dispersal and the Gaussian dispersal kernel [19] that takes the form:
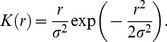
(2)


### Niche model

In real communities, species differ on many aspects. In niche model, we assume species differ in environmental niche optima and the landscape of communities is a simple monotonic environmental gradient which is unaltered in the simulation process. We use monotonic gradients in the model for the sake of simplicity; such gradients may be observed in nature at small spatial scales, or in particular habitats (e.g. vernal pools and steep elevation gradients). Following Schwilk and Ackerly [53] the value of the environmental factor *E* is a linear function of location on the landscape:
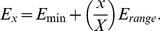
(3)


We equally divide the landscape into *X* small sections and number sections from 0 to *X-1*. *E_x_* is the *E* value of section *x*, *E_min_* and *E_range_* are the minimal value and the range of *E*, respectively.

When there is niche differentiation, the survival probability of species *i* at location *x*, *λ_i,x_*, is associated with environmental factor *E* of location *x*, species optimal environmental condition *μ_i_* and niche breadth *w* [33], as follows:
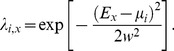
(4)


The niche breadth (*w*) of each species is identical. We label the species from 1 to *M* and the value of the optimal environmental condition of species *i* is [53]:
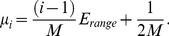
(5)


The probability that a recruit will be of species *i* in a given environment *x* is:
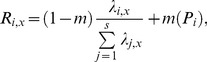
(6)where *s* is the number of species in given dispersal distance.

### Similarity indexes

Repeatability is measured by both the Jaccard similarity index and the Bray-Curtis similarity index. The Jaccard similarity index is based on presence-absence data and is defined as the number of species shared in both communities divided by the number of species in the two communities [37]. It is sensitive to species richness but not to relative species abundance. However, the Bray-Curtis index is much more sensitive to differences in relative abundance, although it is also affected by species richness [38], [39], [54]. Both indices have been widely used in community studies and shown to provide robust estimates of the differences among community structures [37].

### Simulations performed

We repeat the simulations for each community 20 times. For a given community type (either neutral community or niche community) and parameter setting, we examine the repeatability among these replicate communities and then take the average. To investigate the repeatability between neutral and niche communities, and for a given parameter setting, we examine the similarity between 20 neutral replicate communities and 20 niche replicate communities by paired comparison. There are nine immigration rate values with *m* = {10^−8^, 10^−7^, 10^−6^, 10^−5^, 10^−4^, 10^−3^, 10^−2^, 10^−1^, 10^0^} and several dispersal kernels: nearest dispersal, global dispersal and Gaussian dispersal with mean dispersal distance *σ* = {3, 6, 9}. For a given simulation, mean dispersal distance of all individuals is equal. Simulations were performed on lattices with *L* = 120 and an initial species number of *M* = 40. The mortality rate is *d* = 0.1. In niche model, the landscape is equally divided into *X* = 10 sections and the *E_min_* = 0 and *E_rang_* = 1.0. Both the neutral and niche models share the same regional species pool which contains 150 species and the distribution of species relative abundances is a stationary uniform. For niche model, the optimal environmental condition of each species in regional pool is also assigned by equation 5. We perform each simulation for 10^6^ time steps and present the results in the form of a mean and/or a standard deviation. For the Jaccard similarity index and Bray-Curtis similarity index, we use the fossil package [55] implemented in the R version 2.13.1 [56].
