# Correction: Immigration, Local Dispersal Limitation, and the Repeatability of Community Composition under Neutral and Niche Dynamics

**DOI:** 10.1371/annotation/3ca6cdce-4d88-49ec-98f5-1759aed1a122

**Published:** 2012-09-26

**Authors:** Dexiecuo Ai, Philippe Desjardins-Proulx, Chengjin Chu, Gang Wang

There was information omitted from the Funding section. 

The correct Funding statement is: This work was supported by the National Natural Science Foundation of China (No.30970465), the National Natural Science Foundation of China (31000199), Species Fund for Agro-scientific Research in the Public Interest (201203006) and the Fundamental Research Funds for the Central Universities (lzujbky-2010-49). The funders had no role in study design, data collection and analysis, decision to publish, or preparation of the manuscript. 

